# Dentistry and dental care in antiquity: part 2 – Egypt and the Graeco-Roman World

**DOI:** 10.1038/s41415-025-8883-0

**Published:** 2026-01-09

**Authors:** Roger Forshaw

**Affiliations:** KNH Centre for Biomedical Egyptology, Faculty of Biology, Medicine and Health, Oxford Road, The University of Manchester, Manchester, M13 9PL, United Kingdom

## Abstract

This second part of the study on ancient dental care explores dental practices in ancient Egypt and the Graeco-Roman world, drawing on both textual and archaeological evidence to shed new light on dental care and early dentistry. The arid climate and distinctive burial customs of ancient Egypt have resulted in the exceptional preservation of numerous skeletal and mummified remains, allowing researchers to study the full spectrum of dental conditions across three millennia. While a few texts reference medicaments for dental care, there is scant osteological evidence for interventive dental procedures. In contrast, classical Graeco-Roman sources offer richer documentation of dental practices and the evolution of dental care; although, again there is limited archaeological evidence. Early Greek texts, including the *Hippocratic Corpus* and the writings of Aristotle and Theophrastus, lay the groundwork for understanding dental anatomy and basic dental treatment, while later, Roman authors such as Celsus, Pliny and Galen describe surgical extractions, prosthetics and pharmaceutical remedies in greater detail. By integrating these diverse lines of evidence, this analysis highlights both the advancements and limitations of ancient dental care, revealing the complex interplay between culture, diet and medical knowledge.

## Key points

- Offers an insight into ancient dental practices in Egypt and the Classical World.
- Highlights how innovative yet rudimentary remedies and oral hygiene practices, from herbal formulations to possible operative interventions, were used to manage dental issues in antiquity.
- By examining dental care in both ancient Egypt and the Graeco-Roman world, the paper provides a comparative overview of how dental practices evolved over time and influenced modern dentistry.

## Ancient Egypt

**Fig. 1:**
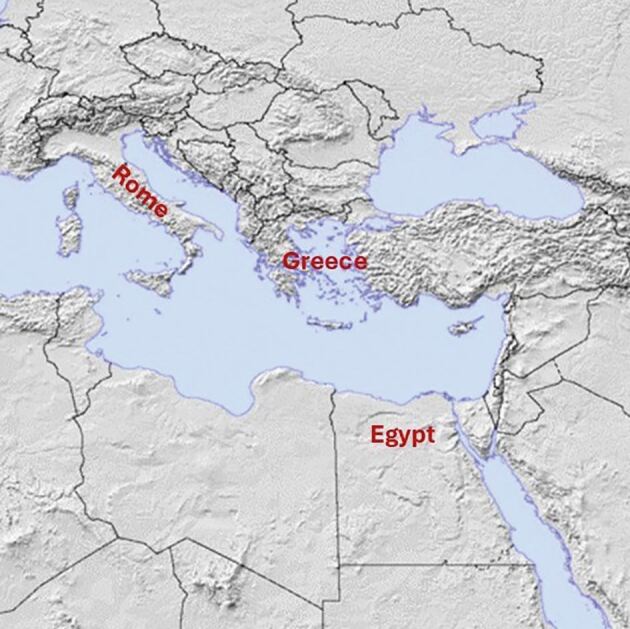
Map of the ancient civilisations. Copyright © 2010, 2013 by Ian Macky

The exceptionally dry climate of ancient Egypt, combined with its unique burial customs, has contributed to the remarkable preservation of numerous human skeletal and mummified remains, surpassing any other civilisation in antiquity (Fig. 1).^1^ Studies of these remains, along with insights derived from surviving documentary and archaeological evidence, have provided a comprehensive understanding of both the pathological and non-pathological conditions affecting the dentitions of the ancient Egyptians.^2^^,^^3^^,^^4^^,^^5^

### Dental pathologies in ancient Egypt

Excessive tooth wear is the most frequent dental condition observed in ancient Egyptian remains and is evident across the civilisation's extensive timeline. It is primarily attributed to a coarse, fibrous diet rendered even more abrasive by the inadvertent inclusion of inorganic particles – mainly wind-blown sand – in their staple food: bread.^6^^,^^7^ In many cases, the tooth wear was so severe that the enamel and dentine rapidly wore away, exposing the pulp chamber, resulting in necrosis of the pulpal tissue and subsequent apical infection. Without dental intervention or antibiotic therapy, such infections could spread systemically and potentially prove fatal.^1^ Langsjoen^8^ judged that dental infections were responsible for numerous deaths in antiquity. In later periods of Egyptian history, the severity of tooth wear decreased, likely due to dietary changes and improved food processing techniques.^9^

Periodontal disease, a significant oral health issue today, was also present in past populations, including ancient Egypt.^10^^,^^11^ Today, it is primarily caused by bacterial irritation from plaque accumulation at the dentogingival junction, a process that appears to have similarly affected ancient peoples.^12^ In contrast, dental caries was infrequently observed.^13^^,^^14^^,^^15^ This low prevalence can be attributed to their diet that lacked fermentable carbohydrates and included abrasive, fibrous foods that naturally minimised plaque retention on tooth surfaces.^16^

### Hygiene practices

Hygiene was important in ancient Egypt, as evidenced by archaeological and textual sources indicating that daily washing was a common practice. Both men and women widely used cosmetics and perfumes. However, there is little evidence of regular oral hygiene routines. It is possible that toothpicks were used, similar to other ancient civilizations where tree twigs, bird feathers, animal bones, and porcupine quills served as oral care implements.^1^ Additionally, priests in ancient Egypt chewed natron pellets, a naturally occurring compound primarily composed of sodium carbonate and sodium bicarbonate, as part of a purification ritual to cleanse their mouths. This practice may have extended to the general population, suggesting an awareness of oral cleanliness despite the lack of explicit evidence for standardised routines.

### Dental profession in ancient Egypt

The existence of a dedicated dental profession in ancient Egypt remains a subject of debate. If there was such a body of professionals, what exactly was their role? Were they operative dental surgeons akin to those of today, or did they primarily prescribe pharmaceutical remedies?^17^^,^^18^^,^^19^ Titles associated with potential dentists and textual references to prescriptions for dental conditions suggest the existence of such a group. However, osteological evidence supporting operative or interventive dentistry is exceedingly rare. Across 3,000 years of pharaonic history, only a few cases have been proposed to support this practice. Unfortunately, these claims are frequently weakened by incomplete documentation of their provenance, imprecise dating, and conflicting later interpretations. Additionally, inaccurate secondary reports have occasionally influenced and further complicated this debate.^20^

One example is a mandible dated to approximately 2500 BC which displays two holes below the roots of an abscessed molar. This was initially interpreted as evidence of ancient dental intervention, suggesting an attempt to alleviate pain by surgically drilling through the mandibular bone to drain the abscess.^20^ However, this interpretation has been challenged, as it would require the ancient Egyptians to possess an understanding of apical periodontitis, an awareness considered unlikely at such an early point in history.^21^ Additionally, the positioning and posterior angulation of the openings would have made such a procedure difficult due to the intervening soft tissues. An alternative and more likely explanation is that the perforations resulted from the natural pathological process, where bone was eroded by pus from an abscess, an occurrence that has been recognised in other ancient specimens. Another possibility is that the openings represent accessory mental foramina, an anatomical variation rather than evidence of surgical intervention.^18^^,^^22^^,^^23^^,^^24^^,^^25^

**Fig. 2:**
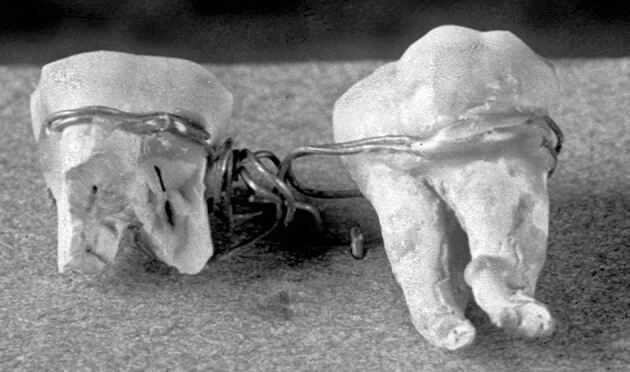
The ‘Giza Bridge'.^18^ Image courtesy of ©Roemer- und Pelizaeus-Museum, Hildesheim

Claims that ancient Egyptians fashioned dental bridges to replace missing teeth or splints to stabilise mobile teeth are intriguing, though largely unsubstantiated. A rare example of what may have been a splint was uncovered in a burial shaft at Giza, near Cairo^26^ (Fig. 2). Initially dated to around 2500 BC, later studies suggest it may instead date to the Ptolemaic period (332–30 BC).^27^ This device, comprising two molar teeth connected by a double strand of thin gold wire, was not found *in situ* but rather in the rubble of the tomb. The delicate nature of the gold wire, along with the possibility that the teeth did not belong to the same individual, suggests that the ‘appliance' was unlikely to have been in use during life. Various interpretations have been proposed regarding its purpose. One theory suggests it was placed in the mummified body to restore wholeness, a practice commonly observed in ancient Egyptian burial customs. Alternatively, the teeth may have functioned as an amulet, possibly believed to provide protection or confer special powers upon the owner.^18^

Another example, excavated from Tura el-Asmant, just south of Cairo, also dated to the Ptolemaic period, was found attached to teeth in a skull, the only one from ancient Egypt to be found *in situ.* This device incorporates a maxillary incisor, possibly the original tooth, used to replace a missing right central incisor. It was secured with silver wire threaded through holes drilled mesio-distally through the crown of the tooth and then connected to the adjacent teeth.^28^ Similar examples of this type of dental work have been documented in Sidon, Greece, and Etruscan cemeteries.^29^

It is not certain if extractions were carried out in ancient Egypt, as although a relatively simple method of relieving toothache, there is no conclusive evidence for the procedure being performed. The textual evidence (medical papyri) are silent on the practice and no instrument has ever been identified as being used for the extraction of teeth. Some researchers have inferred extractions based on the width of alveolar spaces, adjacent tooth inclination and remodelling of alveolar bone in areas of missing teeth.^15^^,^^30^^,^^31^

The question of whether dental restorations were practised in ancient Egypt is also a topic of debate. Recent computerised scans of two Graeco-Roman mummies revealed the presence of extraneous material within grossly carious cavities – these being interpreted as evidence of a primitive form of filling.^32^^,^^33^ However, there is little evidence of tooth preparation, perhaps suggesting that the material may have been placed in the cavities simply to block the ingress of food, rather than as a permanent restorative measure.

**Fig. 3:**
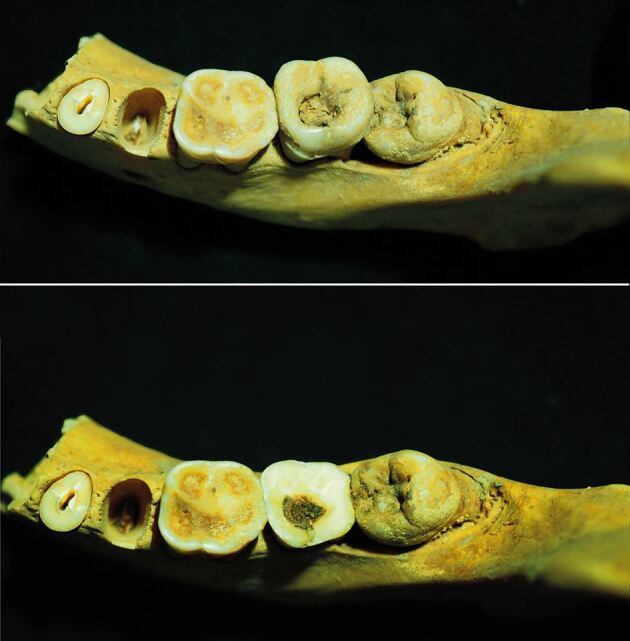
Two views of a possible filling in mandible 298.20.232 from Theban Tomb 298 at Deir el-Medina, Egypt. A small fibrous mass is visible filling the occlusal cavity of the second molar. Image reproduced with permission from Anne Austin

In another example, ‘fibrous material' was discovered in a mandibular molar tooth dated between 1550–1070 BC and found at Deir el-Medina, near Luxor (Fig. 3). The material was suggested to have been intentionally packed into the cavity rather than being accidental debris.^15^ However, fibrous organic substances would normally not have remained intact for long in the oral environment, indicating that this may again represent an attempt to alleviate discomfort from a painful cavity, possibly as a form of self-administered care.

### Pharmaceutical remedies

The most important source of information regarding ancient Egyptian dentistry is found in the medical papyri. These ancient texts, which have survived the ravages of time, include prescriptions for the treatment of oral conditions; although, they remain silent on all operative aspects of dental care.^3^^,^^18^ Of the approximately 18 cases mentioned across various papyri, seven appear to describe remedies aimed at preventing tooth loss by packing various materials in paste form around the teeth and surrounding gums. These pastes were likely intended to harden and serve as a temporary means of stabilising loose teeth. One such example is found in the *Ebers Papyrus* (739):^34^

‘Beginning of the remedies to consolidate a tooth; flour of emmer seeds; ochre; honey; made into a mass; and the tooth to be fattened therewith'.

This formulation may have had some medicinal value, as ochres (iron oxides) possess mild astringent and antiseptic properties, while honey is hypertonic and can eradicate microorganisms by drawing water out of them through osmosis.^35^ Thus, honey would likely have inhibited bacterial growth and helped reduce inflammation in infected gingival and mucosal tissues. Another possibility is that the material was used to fill carious cavities; although, the incidence of caries in ancient Egypt was relatively low, while periodontal disease was more prevalent.

**Fig. 4:**
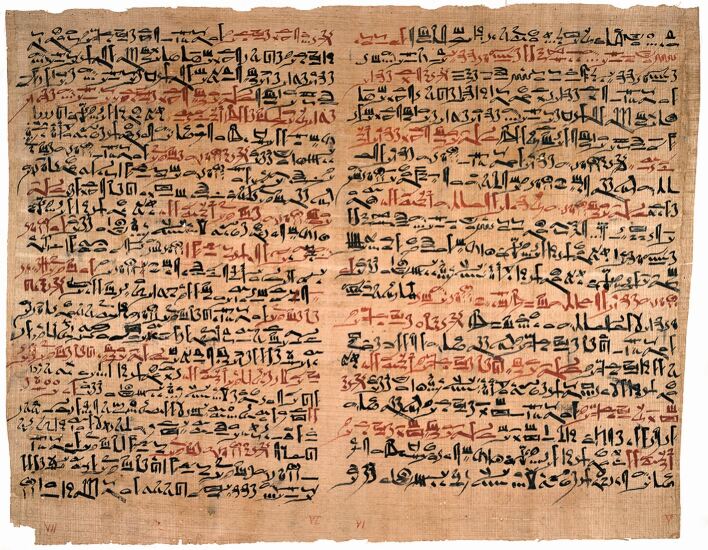
*Edwin Smith Papyrus*, recto columns six and seven, describing a method for correcting a dislocated mandible. Image courtesy of the National Library of Medicine/Science Photo Library

Additionally, the texts describe various remedies for oral ulcers, abscesses, and gingival infections, some of which may have offered some limited short-term relief. Also included are prescriptions for mouthwashes, as well as methods for treating fractures of the maxilla and mandible. Notably, there are instructions for managing a dislocated jaw using a technique similar to that practised today^36^ (Fig. 4).

Thus, although evidence suggests that ancient Egyptians experienced painful dental conditions, such as severe tooth wear, abscesses, and periodontal disease, the treatments available at the time likely offered only limited and temporary relief.

## The Classical World: Graeco-Roman dentistry

### Greece

#### Ancient Greek dental practices and textual evidence

Again, ancient texts serve as the primary source of information on dental care in the Graeco-Roman world, as archaeological evidence of any possible dentistry is limited (Fig. 1). A particularly valuable source is the *Hippocratic Corpus*, a collection of ancient Greek manuscripts, some of which are attributed to the 5^th^ century BC physician Hippocrates, while others were authored by his associates and later scholars.^37^^,^^38^ Included within these medical works are aspects of dental anatomy, which include references to a tooth numbering system resembling the one in use today, as well as probably the earliest references to the term ‘wisdom teeth'. They also describe operative procedures, including extractions, abscess incisions, and the treatment of mandibular dislocations and fractures. Splinting with gold wire was recommended for displaced or mobile teeth, and other passages addressed conditions such as dental caries, gingivitis and osteomyelitis.^39^

**Fig. 5:**
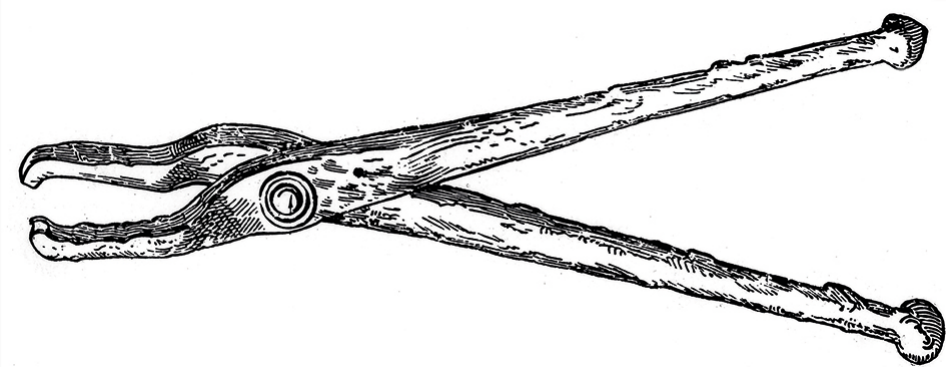
A pair of 2^nd^-century AD Greek forceps discovered in Lidoriki, Greece. Image source: Wellcome Collection

The writings of Aristotle (384–322 BC), the eminent Greek philosopher, encompassed a wide range of topics – among them, the natural sciences.^40^ Although he discussed dental anatomy, his therapeutic recommendations were limited, addressing only the use of iron dental forceps for extractions (Fig. 5). References to oral hygiene are largely absent in these early texts and did not become a notable topic of discussion until Greece became a Roman province in 146 BC.

#### Botanical remedies and philosophical contributions

In his book *The Enquiry into Plants*, Theophrastus (c. 287–371 BC), a close associate of Aristotle and often regarded as the ‘father of botany,' offered useful insights on plant functions and properties. His work identified narcotic plants that could alleviate pain, observations that were later reiterated by other authors.^41^

Pedanius Dioscorides (c. AD 40–90), a Greek physician, further advanced the field with his seminal five-volume encyclopedia *De Materia Medica*, which focused on herbal medicine and medicinal substances.^42^ This text details numerous remedies for toothache, such as cedar decoctions and the insertion of lizard liver into carious cavities, many of which were likely ineffective. However, for periodontal disease, Dioscorides recommended plant-based mouthwashes with styptic properties, including olive oil and pomegranate leaves, constituents which may have helped to reduce inflammation. Abrasive substances like oyster shells and ground deer antlers were prescribed to clean teeth, and various preparations were suggested to address halitosis.^43^

### Rome

With the expansion of the Roman Empire, certain Greek slaves were brought to Rome to serve as physicians. Over time, many gained their freedom and began to influence the development of Roman medicine. During the first two centuries of the Roman Empire and beyond, Greek physicians dominated the medical field and were well-regarded as competent practitioners. Dentistry, however, at this time did not exist as a distinct profession but was instead integrated into general medical practice.

#### Textual evidence

Ancient texts continue to provide valuable insights into historical dental practices. Aulus Cornelius Celsus (c. 25 BC-AD 50), a Roman encyclopedist, is best known for his medical treatise, *De Medicina,* which includes a section on tooth extraction.^44^ He described a meticulous technique for carefully removing tooth and root fragments, not dissimilar to descriptions found in modern textbooks. He advised extracting deciduous teeth when their permanent successors began erupting prematurely, and outlined techniques for stabilising loose teeth and smoothing fractured teeth that irritated soft tissues. Additionally, he recommended cauterising the gingiva of periodontally compromised teeth with a heated iron, a practice intended to control infection and inflammation.

*De Medicina* also covers a wide range of pharmaceutical treatments for oral pain. Celsus advocated the use of medicaments to manage painful conditions of the gingiva and oral mucosa, reserving extraction as a last resort. His recommended remedies included cinquefoil, now recognised for its wound-healing and anti-inflammatory properties, and the root of mandrake, known for its potent narcotic and sedative effects.^45^^,^^46^ Regarding periodontal disease, Celsus advised, ‘if the gums separate from the teeth, it is beneficial to chew purslane or pears and apples and keep their juices in the mouth'.^47^ His contemporary, Scribonius Largus (0–AD 50), also documented numerous dental remedies and analgesics among his many prescriptions.^48^

Pliny the Elder (AD 23–79), in his work, *Natural History*, also described numerous remedies for oral and dental ailments.^49^ He recommended the ashes of deer antlers to reduce tooth mobility and alleviate dental pain. For cleaning teeth, he again advocated the use of antler ashes but now mixed with powdered deer antlers. Additionally, he suggested various animal-derived products, including substances from oxen, mice and sparrows as treatments for oral pain.

Galen of Pergamon (c. AD 129–216), a seminal figure in ancient medicine, was a physician, researcher and philosopher. His extensive writings included detailed anatomical descriptions of the teeth and oral cavity. By synthesising earlier Greek medical traditions, he laid the foundation for integrating dental care into broader health practices, a legacy that shaped medical thought well into the Middle Ages and beyond.^50^^,^^51^

#### Archaeological evidence of dental operative procedures

Excavations near the Temple of Castor and Pollux, in the Roman Forum, have provided physical evidence of dental extractions. A drain within a small commercial establishment was found to contain 86 human teeth (primarily molars), all with extensive carious lesions, many of which extended as far as the pulp chamber.^52^^,^^53^ Several molars bore distinct horizontal tool marks in the interproximal cervical region, possibly caused by a metal elevator used for extraction.^54^

Additional findings, including glass containers with traces of cosmetics and medicine, ointment jars, and pharmaceutical artifacts, suggest that the establishment may have functioned as both a pharmacy and a premises for dental extractions. This highlights the interconnected nature of medicine, pharmacy and cosmetics in Roman society.^55^^,^^56^

**Fig. 6:**
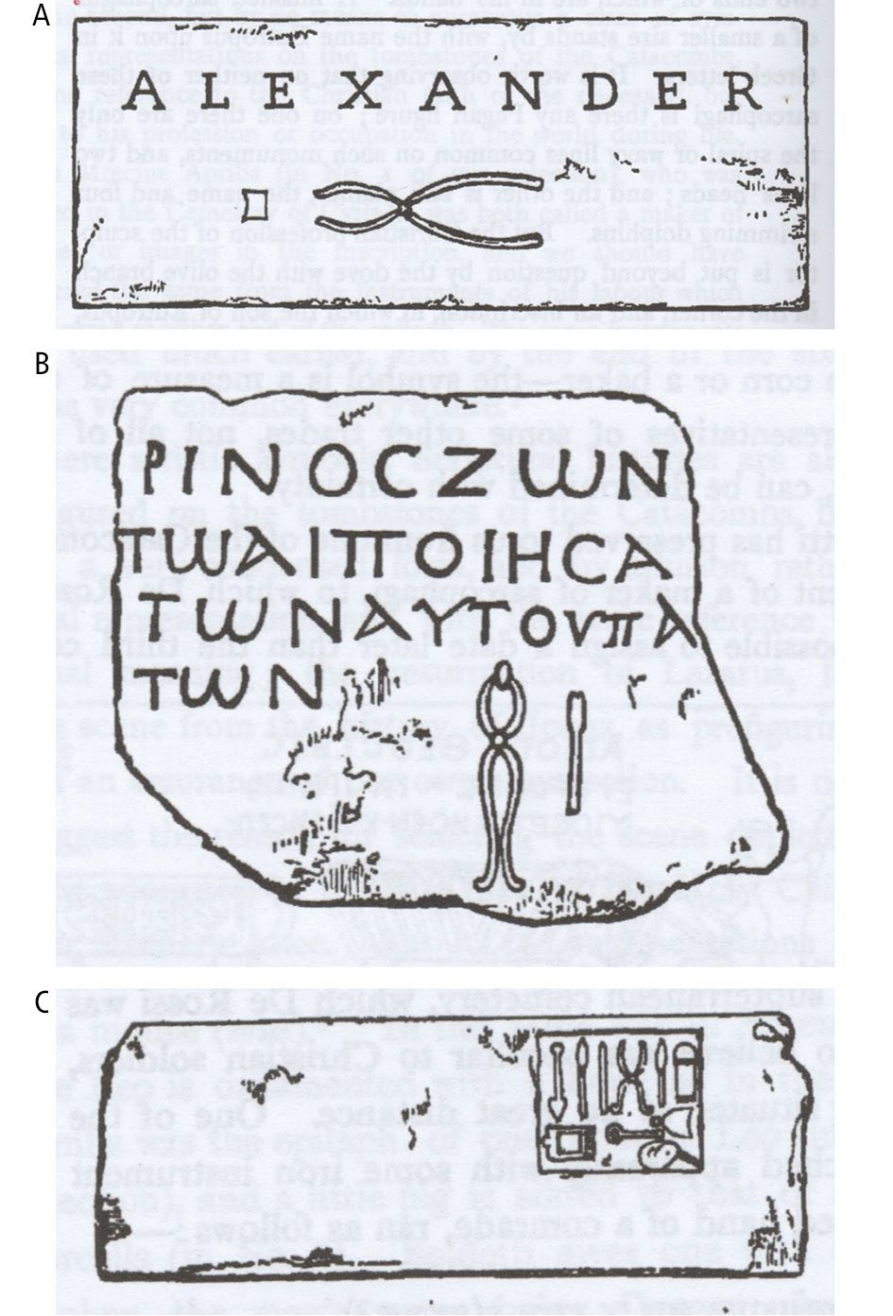
(A, B, C) Possible depictions of dental forceps inscribed on Roman stone funerary monuments. (A) Gravestone from the cemetery of Calpodius. The square to the left of the forceps is thought to represent a tooth, with the whole inscription interpreted as ‘Alexander the Dentist'. (B) Gravestone of a possible dentist found near the Basilica of San Lorenzo. One of the two instruments appears to depict dental forceps clasping a tooth. (C) A funerary relief depicting an assortment of surgical instruments, possibly including a pair of dental forceps^57^

Further evidence supporting the practice of dental extractions in ancient Rome is found inscribed on funerary monuments, where depictions of instruments believed to be dental forceps have been identified (Fig. 6A, Fig. 6B). One notable relief, commemorating an unknown individual, features an assortment of surgical tools, suggesting that he may have been a surgeon. Among the instruments is a pair that closely resembles ancient dental forceps^57^ (Fig. 6C). Although numerous collections of implements, often interpreted as medical instruments, have been excavated at various sites, their precise function often remains uncertain due to the lack of explanatory references and definitive contextual evidence.

#### Dental prosthesis and oral health in ancient Rome

**Fig. 7:**
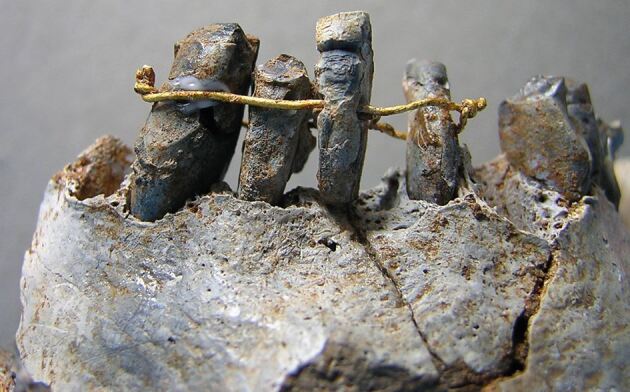
A partially cremated Roman dental prosthesis dating to 1^st^–2^nd^ century AD. Image reproduced with permission from Simona Minozzi

Although ancient Roman texts reference dental prostheses, archaeological evidence remains scarce. However, a significant exception was discovered at the Collatina Necropolis, dating to the 1^st^–2^nd^ century AD (Fig. 7). This device, designed to replace missing central incisors, featured a gold wire looped around the mandibular anterior teeth as a retainer. One of the pontics, likely the original incisor, was perforated to accommodate the wire, with its root apex trimmed to gum fit the appliance.^58^

Several studies have examined dental pathology, with Manzi's research on two Roman cemeteries (1^st^–3^rd^ century AD) revealing oral health disparities between social groups.^55^^,^^59^^,^^60^ At Isola Sacra, a middle-class cemetery, lower incidences of caries, dental abscesses, tooth wear and periodontal disease were observed compared to the site of Lucus Feroniae, a rural cemetery primarily associated with enslaved individuals. These differences align with expectations, as living conditions would likely have influenced oral health outcomes. Dental surveys from Pompeii and Herculaneum, the Roman cities buried by the eruption of Mount Vesuvius in AD 79, indicate relatively low levels of caries.^61^ This trend has been attributed to the high fluoride content in the local drinking water, which reached several thousand parts per million, a concentration that persists to this day.^62^

A study of skeletal remains of individuals believed to have been part of the labour force at a villa in Vallerano, on the outskirts of Rome, during the 2^nd^–3^rd^ century AD, further illustrates the impact of socioeconomic conditions on dental health.^63^ While the prevalence of carious teeth, periapical defects and antemortem tooth loss was low, a high incidence of linear enamel hypoplasia was recorded, suggesting episodes of nutritional and physiological stress during early development. Additional skeletal evidence, including hypoplastic abnormalities and indications of parasitic infections, further reflects the harsh living conditions experienced by these individuals.

## Conclusion

Textual and archaeological evidence from the Graeco-Roman world suggests a higher level of dental knowledge in antiquity than previously assumed. While some treatments may have been effective, many prescribed remedies for dental ailments likely offered limited or no therapeutic benefit. Collectively, these findings not only reflect the ingenuity of ancient practitioners but also highlights the challenges they faced with the resources available to them.
